# The *Caenorhabditis elegans* Synthetic Multivulva Genes Prevent Ras Pathway Activation by Tightly Repressing Global Ectopic Expression of *lin-3* EGF

**DOI:** 10.1371/journal.pgen.1002418

**Published:** 2011-12-29

**Authors:** Adam M. Saffer, Dong Hyun Kim, Alexander van Oudenaarden, H. Robert Horvitz

**Affiliations:** 1 Department of Biology, Massachusetts Institute of Technology, Cambridge, Massachusetts, United States of America; 2 Howard Hughes Medical Institute, Massachusetts Institute of Technology, Cambridge, Massachusetts, United States of America; 3 Department of Physics, Massachusetts Institute of Technology, Cambridge, Massachusetts, United States of America; University of California San Diego, United States of America

## Abstract

The *Caenorhabditis elegans* class A and B synthetic multivulva (synMuv) genes redundantly antagonize an EGF/Ras pathway to prevent ectopic vulval induction. We identify a class A synMuv mutation in the promoter of the *lin-3* EGF gene, establishing that *lin-3* is the key biological target of the class A synMuv genes in vulval development and that the repressive activities of the class A and B synMuv pathways are integrated at the level of *lin-3* expression. Using FISH with single mRNA molecule resolution, we find that *lin-3* EGF expression is tightly restricted to only a few tissues in wild-type animals, including the germline. In synMuv double mutants, *lin-3* EGF is ectopically expressed at low levels throughout the animal. Our findings reveal that the widespread ectopic expression of a growth factor mRNA at concentrations much lower than that in the normal domain of expression can abnormally activate the Ras pathway and alter cell fates. These results suggest hypotheses for the mechanistic basis of the functional redundancy between the tumor-suppressor-like class A and B synMuv genes: the class A synMuv genes either directly or indirectly specifically repress ectopic *lin-3* expression; while the class B synMuv genes might function similarly, but alternatively might act to repress *lin-3* as a consequence of their role in preventing cells from adopting a germline-like fate. Analogous genes in mammals might function as tumor suppressors by preventing broad ectopic expression of EGF-like ligands.

## Author Summary

Extracellular signals that drive cells to divide must be carefully restricted so that only the correct cells receive those signals. Failure to properly control the expression of signaling molecules can lead to aberrant development and cancer. Studies of vulval development in the nematode *Caenorhabditis elegans* have helped define various multi-step signaling pathways involved in cancer. Here we report that two groups of proteins that control the EGF/Ras/MAP kinase pathway of vulval development act by tightly repressing the spatial expression of the gene *lin-3*, which encodes an EGF-like signaling molecule. Using a technique that detects single mRNA molecules, we show that inactivation of these proteins causes a low ectopic expression of *lin-3* in many cells. In response, the EGF/Ras/MAP kinase pathway is activated in cells normally not exposed to the *lin-3* signal, and vulval development is abnormal. This process is analogous to the cancerous growth that occurs in humans when mutations cause both tumor cells and the microenvironment surrounding the tumor cells to ectopically express factors that drive cellular proliferation. We propose that mammalian genes analogous to those that repress *lin-3* expression in *C. elegans* vulval development act as tumor suppressors by preventing broad ectopic expression of EGF-like ligands.

## Introduction

Signaling by epidermal growth factor (EGF) family ligands and EGF receptor (EGFR) family tyrosine kinases controls many aspects of mammalian development and can drive cancers: EGFRs are commonly overexpressed or constitutively activated by mutations in tumor cells [1], and EGF-family ligands can be misregulated in cancer. For example, the EGF-family ligands heparin-binding EGF-like growth factor, amphiregulin, and TGF-α are upregulated in cancer cells from many different cancer types [2], [3], and TGF-α overexpression causes widespread epithelial hyperplasia in mice [4], [5]. Growth factors often signal through a Ras pathway, and approximately 20% of tumors carry a constitutively active Ras mutation [6].

In the nematode *Caenorhabditis elegans* the EGF-family ligand LIN-3 acts through the EGFR LET-23 and the Ras protein LET-60 to control many aspects of development, including the induction of the hermaphrodite vulva [7]–[10]. In wild-type animals, of a set of six equipotent cells, three (P5.p, P6.p and P7.p) adopt vulval cell fates, while the other three (P3.4, P4.p, and P8.p) adopt non-vulval fates [11]. The expression of vulval cell fates requires EGF/Ras signaling, and mutations that reduce EGF/Ras signaling cause a vulvaless (Vul) phenotype in which none of the six cells adopts vulval cell fates [7]–[10]. The anchor cell, located closest to P6.p, is the only cell that both expresses LIN-3 EGF and is located near the six Pn.p cells [10], and laser ablation of the anchor cell results in a Vul phenotype [12] like that seen in mutants defective in *lin-3* EGF or *let-23* EGFR. Overactivation of the EGF/Ras pathway, by overexpression of *lin-3* EGF or by an activating mutation in either *let-23* EGFR or *let-60* Ras, causes a multivulva (Muv) phenotype in which all six Pn.p cells adopt vulval cell fates [8]–[10],[13].

In vulval development, EGF/Ras signaling is antagonized by the synthetic multivulva (synMuv) genes. The synMuv genes define two classes, A and B [14], [15]. In synMuv single mutants or in class A double mutants or class B double mutants, vulval development is mostly normal. By contrast, animals mutant in both a class A synMuv gene and a class B synMuv gene exhibit a strong Muv phenotype. Many class B synMuv genes have homologs that function in histone modification, chromatin remodeling, and transcriptional repression. For example, the class B synMuv genes encode a DP/E2F/Rb complex [16], [17], a nucleosome remodeling and deacteylase (NuRD) complex [18], [19], two histone methyltransferases [20], [21] and a heterochromatin protein 1 homolog [22]. Of the three molecularly-characterized class A synMuv genes, two encode proteins with a zinc-finger-like THAP domain [23]–[25]. The expression patterns of three class A synMuv proteins have been studied, and all three are localized to the nucleus, suggesting that class A synMuv proteins regulate transcription [25], [26].

The synMuv genes function at least in part by repressing expression of *lin-3* EGF. Loss-of-function mutations in either *let-23* EGFR or *lin-3* EGF can suppress the synMuv phenotype [16], [27], [28], indicating that the synMuv genes act upstream of or in parallel to *lin-3*. Furthermore, *lin-3* mRNA levels are increased in synMuv double mutants but not in synMuv single mutants [27], and overexpression of *lin-3* EGF causes a Muv phenotype [10].

Laser ablation of the anchor cell, the source of LIN-3 in wild-type vulval development, does not fully suppress the Muv phenotype of synMuv double mutants [28], indicating that synMuv genes cannot simply prevent overexpression of *lin-3* from the anchor cell. Mosaic analyses of the class B synMuv gene *lin-37* and the *lin-15* locus, which contains both a class A and a class B synMuv gene, did not identify a single site of action. Both experiments indicated that *lin-15* and *lin-37* do not act cell-autonomously in the Pn.P cells and suggested that *lin-15* and *lin-37* might function in the syncytial hypodermal cell hyp7 [29], [30]. Heterologous expression experiments showed that the class B synMuv gene *lin-35* functions in hyp7 to antagonize vulval cell fates, and tissue-specific RNAi of *lin-3* in hyp7 can suppress the synMuv phenotype, indicating that repression of *lin-3* in the hypoderm is an important function of the synMuv genes [27], [31]. Another study using the same heterologous promoters found that the class B synMuv gene *hpl-2* functions in both hyp7 and the Pn.p cells [32]. However, it is not known where *lin-3* is overexpressed in synMuv mutants, how the synMuv genes control *lin-3* expression, or if the synMuv genes control targets other than *lin-3* important for vulval development.

Here we report the identification of a *lin-3* EGF promoter mutation that causes a dominant class A synMuv phenotype. The effect of this mutation reveals that the only major role of the class A synMuv genes in vulval development is to repress *lin-3*. We find that *lin-3* mRNA is ectopically expressed throughout the animal in synMuv mutants. Our results show that low levels of ectopic *lin-3* expression outside the cells that normally produce and respond to *lin-3* can adversely alter the development of *C. elegans*, and we propose that the class A and class B synMuv genes might prevent ectopic *lin-3* expression by distinct mechanisms.

## Results

### *n4441* causes a dominant class A synMuv phenotype

During a screen for new class A synMuv mutations, we identified a Muv animal in the F_1_ generation after ethyl methanesulfonate (EMS) mutagenesis of the class B synMuv mutant *lin-52(n771)*. We named the mutation that caused this defect *n4441*. To seek additional mutations that like *n4441* dominantly cause a class A synMuv phenotype, we screened approximately 492,000 F_1_ progeny of *lin-52(n771)* animals mutagenized by EMS and approximately 89,000 progeny of animals mutagenized by N-ethyl-N-nitrosourea (ENU), but we did not identify any additional class A synMuv mutants. As a single mutant, *n4441* animals are wild-type at 20°C and exhibit a low penetrance Muv defect at 25°C (Table 1), comparable to that of most class A synMuv mutants [15]. Double mutants between *n4441* and the class B synMuv mutations *lin-15B(n744)*, *lin-52(n771)*, or *lin-61(n3447)* exhibit a strong synMuv phenotype. *n4441* causes a fully penetrant Muv defect as a heterozygote in the class B synMuv mutant background *lin-15B(n744)*, indicating that *n4441* dominantly causes a class A synMuv phenotype. *n4441* causes a 97% penetrant synMuv defect in the weak class B synMuv mutant background *lin-61(n3447)* at 22.5°C, comparable to the previously reported phenotype of double mutants between *lin-61(n3447)* and the strong class A synMuv mutations *lin-15A(n767)* or *lin-38(n751)* [15].

**Table 1:** *lin-3(n4441)* causes a dominant class A synMuv phenotype.

genotype	% multivulva ± s.d. (n)^a^			
	20°C		25°C	
N2	0±0	(1422)	0±0	(1368)
*lin-3(n4441)*	0±0	(954)	1±0.4	(1105)
*lin-3(n4441)/+* b	0±0	(621)	1±2	(830)
*lin-15B(n744)*	0±0	(1058)	0±0	(642)
*lin-3(n4441); lin-15B(n744)*	100±0	(729)	100±0	(248)
*lin-3(n4441)/+; lin-15B(n744)* c	100±0	(795)	100±0	(152)
*lin-52(n771)*	0±0	(1029)	0±0	(1233)
*lin-3(n4441); lin-52(n771)*	98±0.1	(974)	100±0	(1244)
*lin-61(n3447)*	0±0	(1039)	0±0	(986)
*lin-61(n3447); lin-3(n4441)*	17±13	(778)	100±0	(1201)
*mys-1(n3681)*	0.4±0.4	(833)	Lva^d^	
*mys-1(n3681); lin-3(n4441)*	56±9	(640)	Lva^d^	
*lin-8(n2731)*	0±0	(902)	0±0	(910)
*lin-8(n2731); lin-3(n4441)*	0.4±0.5	(1093)	1±2	(788)
*lin-15A(n767)*	0.1±0.2	(1022)	2±1	(1009)
*lin-3(n4441); lin-15A(n767)*	5±3	(831)	60±17	(609)
*lin-38(n751)*	0±0	(947)	1±1	(1307)
*lin-38(n751); lin-3(n4441)*	1±2	(1028)	12±3	(889)
*lin-56(n2728)*	0±0	(932)	1±1	(733)
*lin-56(n2728); lin-3(n4441)*	0.3±0.3	(930)	6±4	(843)

a Animals were scored as Muv if any ventral ectopic protrusions were observed. For each strain, animals were scored on three separate days, and % multivulva shown is the average for those three days. SD, standard deviation. n, total number of animals scored.

b These animals were also heterozygous for *dpy-17(e164)* and *unc-32(e189)* and descended from *lin-3(n4441)* homozygous parents.

c These animals were also heterozygous for *dpy-5(e61)* and descended from *dpy-5(e61); lin-3(n4441); lin-15B(n744)* parents.

d Lva, larval arrest. Animals arrested as larvae, precluding the assaying of vulval development.

To determine how *n4441* interacts with other class A synMuv mutations, we built double mutants between *n4441* and an allele of each known class A synMuv gene. We used the putative null alleles *lin-8(n2731)*, *lin-15A(n767)*, and *lin-56(n2728)* and the missense allele *lin-38(n751)*, since a null allele of *lin-38* causes lethality (A.M.S and H.R.H., unpublished results). At 20°C and 25°C, the double mutants *n4441; lin-15A(n767)*, *lin-38(n751); n4441*, and *lin-56(n2728); n4441* were enhanced for the Muv phenotype when compared to their respective single mutants (Table 1). The *lin-8(n2731); n4441* double mutant was roughly comparable to *n4441* alone when scored at 25°C and also exhibited a low penetrance Muv defect at 20°C, which neither *n4441* or *lin-8(n2731)* did on their own (Table 1). Thus, mutations in all known class A synMuv genes can enhance the Muv phenotype of *n4441*, but the enhancement is much weaker than the enhancement caused by class B synMuv mutations.

Several members of a Tip60/NuA4 histone acetyltransferase complex were previously identified as class C synMuv genes [33]. Class C synMuv genes are strongly Muv in combination with class A synMuv mutations and weakly Muv in combination with class B synMuv mutations and can be considered a subset of the class B synMuv genes [15]. To test if *n4441* might be a class C synMuv gene, we built a double mutant between *n4441* and the partial loss-of-function class C synMuv mutation *mys-1(n3681)*, as null mutants of *mys-1* cannot be maintained as homozygous strains [33]. The *mys-1(n3681); n4441* double mutant exhibited a 56% penetrant Muv defect at 20°C, which is much stronger than the 5% penetrant Muv defect of the *n4441; lin-15A(n767)* strain at 20°C, despite the fact that *lin-15A(n767)* is a null mutation. We conclude that *n4441* is not a class C synMuv mutation.

### *n4441* is an allele of *lin-3*

**Figure 1:**
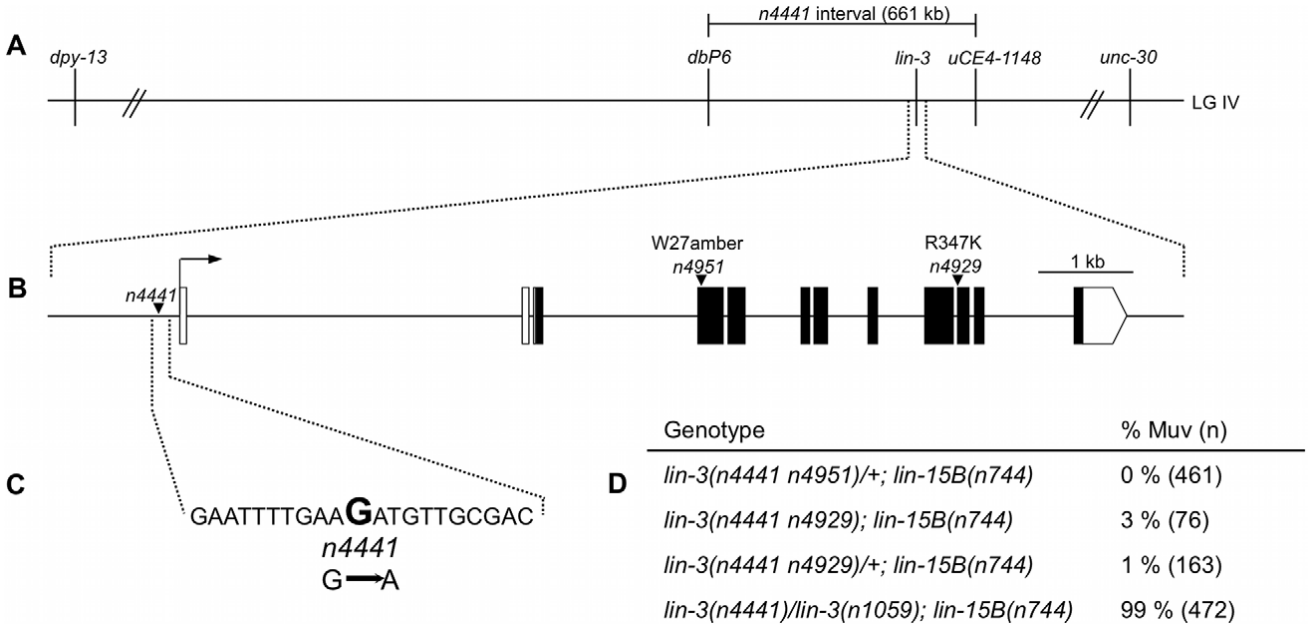
*n4441* is an allele of *lin-3*. (A) Genetic map showing *n4441* on LGIV. *n4441* was localized between *dpy-13* and *unc-30* by three-factor mapping. SNP mapping using polymorphisms present in the CB4856 strain further localized *n4441* to a 661 kb region between the SNPs *dbP6* at 10909553 and *uCE4-1148* at 11570158 of LGIV (data not shown). (B) The *lin-3* locus. The *lin-3a* isoform (Wormbase web site, http://www.wormbase.org, release WS200, Mar 20 2009) is shown. Solid boxes, exons; open boxes, UTRs. The start of the transcript is indicated by an arrow. Arrowheads indicate the locations of mutations. *n4951* is a nonsense mutation that truncates LIN-3 after 26 amino acids, and *n4929* is a missense mutation that converts an arginine to a lysine at amino acid 347. (C) *n4441* is a G-to-A mutation at nucleotide 30904 of cosmid F36H1, 211 bp upstream of the *lin-3* transcript. No other mutations were present in *n4441* mutants in the region shown in (B). (D) A *lin-3* loss-of-function mutation suppresses the *n4441* synMuv phenotype in *cis* but not in *trans*. *lin-3(n1059)* is a nonsense mutation in *lin-3* [37]. *lin-3(n4441 n4951)* and *lin-3(n4441 n4929)* heterozygotes also carried the *nT1[qIs51]* translocation. All animals were grown at 20°C. Animals were scored as Muv if any ventral ectopic protrusions were observed. n, total number of animals scored.

By performing SNP mapping experiments using the CB4856 polymorphic strain of *C. elegans*, we mapped the *n4441* mutation to a 661 kb region containing approximately 170 genes between SNPs *dbP6* and *uCE4-1148* (Figure 1A). *n4441* dominantly causes a synMuv phenotype and thus might well be a gain-of-function mutation, so we sought loss-of-function mutations in the gene affected by *n4441*. *n4441/nT1[qIs51]; lin-15B(n744)* animals, which display a fully penetrant Muv defect, were mutagenized with EMS. The *nT1[qIs51]* translocation causes inviability when homozygous and suppresses recombination across an interval that includes *lin-3* [34]. Approximately 6,800 F_1_ progeny were screened, and two animals were identified that were non-Muv and produced only non-Muv progeny, indicating that they contained a suppressor mutation tightly linked to *n4441*. We named these mutations *n4929* and *n4951*. *n4441 n4929; lin-15B(n744)* animals were sterile and exhibited a very low penetrance Muv defect (Figure 1D). *n4441 n4951/nT1[qIs51]* animals were superficially wild-type with no Muv defect, and *n4441 n4951* homozygotes died as L1 larvae with a rod-like appearance (Figure 1D). The rod-like lethal phenotype is characteristic of loss-of-function mutations in genes in the EGF/Ras pathway required for vulval induction [35]. The only known gene in the EGF/Ras pathway in the genetic interval containing *n4441* is *lin-3*, which encodes the EGF ligand. Strong loss-of-function alleles of *lin-3* cause a rod-like lethal phenotype, and *lin-3* mutations can also cause sterility [36], [37]. The *n4929* mutant carries a G-to-A transition in the first nucleotide of exon 8 of *lin-3* and is predicted to mutate an arginine to a lysine at amino acid 347 of LIN-3 (Figure 1B). The *n4951* mutant carries a G-to-A transition that results in a nonsense mutation predicted to truncate LIN-3 after only 26 amino acids, before the EGF domain (Figure 1B). The *lin-3(n1059)* nonsense mutation failed to complement the sterility caused by *n4929* and the lethality caused by *n4951*, proving that *n4929* and *n4951* are alleles of *lin-3*. Since the *lin-3(n4951)* nonsense mutation suppressed the *n4441* synMuv defect in *cis*, but the *lin-3(n1059)* nonsense mutation did not suppress the *n4441* synMuv defect in *trans* (Figure 1D), a *lin-3* loss-of-function mutation is a *cis* dominant suppressor of *n4441*, indicating that *n4441* is a gain-of-function allele of *lin-3*.

We determined the sequences of all exons and introns of *lin-3* and of approximately 11 kb of upstream DNA in *lin-3(n4441)* mutants. The only mutation was a G-to-A transition at nucleotide 30904 of cosmid F36H1, approximately 200 bp upstream of the *lin-3* transcript *F36H1.4a* (http://www.wormbase.org, release WS200, 20 Mar 2009) (Figure 1C). To show that the F36H1(30904) mutation is required for the class A synMuv phenotype caused by *lin-3(n4441)*, we sought recombinants between *lin-3(n4441)* and the *lin-3(n4951)* nonsense mutation, which is 5.3 kb downstream of F36H1(30904). We screened approximately 90,000 progeny from *lin-3(n4441 n4951)/+; lin-15B(n744)* animals, identified five independent Muv animals and established homozygous lines. None of the five lines contained the *lin-3(n4951)* mutation, and all five carried the F36H1(30904) G-to-A mutation. Thus, the *lin-3(n4441)* mutation that causes the class A synMuv phenotype must be to the left of *lin-3(n4951)*, because if it were to the right then the recombinants would not carry the F36H1(30904) mutation. The 5.3 kb between F36H1(30904) and *lin-3(n4951)*, as well as 10.8 kb of DNA upstream of F36H1(30904), carried no additional mutations in *lin-3(n4441)* animals. If the mutation that causes the *lin-3(n4441)* synMuv phenotype is not the F36H1(30904) mutation, then the *lin-3(n4441)* mutation must be at least 10.8 kb to the left of the F36H1(30904) mutation. However, in that case, assuming a constant recombination rate throughout the *lin-3* interval, the likelihood that all five recombination events would have occurred between F36H1(30904) and *lin-3(n4951)* is ((5.3)/(5.3+10.8))^5^, or <0.004. We conclude that the G-to-A mutation at nucleotide 30904 of cosmid F36F1 is necessary for the class A synMuv phenotype caused by *lin-3(n4441)*. However, we cannot rule out the possibility there is a second mutation more than 11 kb upstream of *lin-3* that is also required along with the F36H1(30904) mutation to cause a class A synMuv phenotype. There are no known consensus transcription factor binding sites that include the site of the *lin-3(n4441)* mutation (Transfac database of known transcription binding sites; http://www.gene-regulation.com). The region surrounding the *lin-3(n4441)* mutation is moderately conserved in the related nematodes *C. briggsae* and *C. remanei* (data not shown).

*lin-3(n4441)* might be a class A synMuv specific allele of *lin-3*. Alternatively, *lin-3(n4441)* might cause weak overexpression of *lin-3* if weak overexpression of *lin-3* behaves like a class A synMuv mutation. To differentiate between these alternatives, we overexpressed *lin-3* weakly using the *syIs12* integrated transgene. *syIs12* expresses the EGF domain of *lin-3* under the control of a heat-shock promoter [38]. At 20°C in the absence of heat-shock, *syIs12* did not cause a Muv phenotype (Table 2). *syIs12; lin-15B(n744)* animals were mostly wild-type, with only a 1% penetrant Muv defect, whereas *syIs12; lin-15A(n767)* animals exhibited a Muv defect with 40% penetrance (Table 2). Thus, weak overexpression of *lin-3* from the *syIs12* transgene was enhanced by a class A synMuv mutation but not by a class B synMuv mutation. By contrast, *lin-3(n4441)* was enhanced much more strongly by class B synMuv mutations than by class A synMuv mutations (Table 1). We conclude that *lin-3(n4441)* is a class A synMuv specific allele of *lin-3* and does not simply cause weak overexpression of *lin-3*.

**Table 2:** *lin-3* overexpression is enhanced more strongly by a class A synMuv mutation than by a class B synMuv mutation.

genotype	% multivulva (n)^a^
*syIs12* b	0 (92)
*syIs12; lin-15A(n767)*	38 (105)
*syIs12; lin-15B(n744)*	1 (100)

a Animals were scored as Muv if any ventral ectopic protrusions were observed. n, total number of animals scored.

b *syIs12* is an integrated transgene expressing the *lin-3* EGF domain under the control of a heat-shock promoter [38]. Animals were assayed in the absence of heat-shock.

### *lin-3(n4441)* specifically prevents repression of *lin-3*

**Figure 2:**
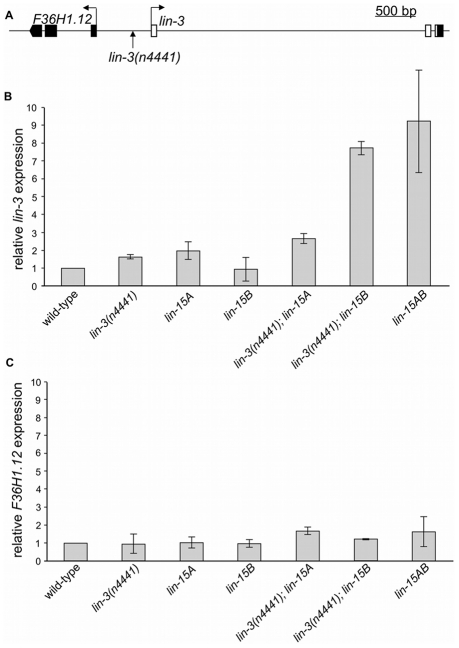
The *lin-3(n4441)* mutation specifically prevents repression of *lin-3*. (A) The *lin-3(n4441)* mutation is located 211 bp upstream of the *lin-3* transcript. The gene *F36H1.12* is upstream of *lin-3* in the opposite orientation, and *lin-3(n4441)* is located 465 bp from the predicted *F36H1.12* transcript. Solid boxes, exons; open boxes, UTRs. (B) *lin-3* mRNA levels in *lin-3(n4441)* single and double mutants. As reported previously [27], *lin-3* mRNA levels are substantially increased in *lin-15AB* double mutants but not in *lin-15A* or *lin-15B* single mutants. Like other class A synMuv mutations, *lin-3(n4441)* caused a substantial increase in *lin-3* mRNA levels only in a class B synMuv mutant background. Realtime RT-PCR experiments were performed using RNA harvested at the late L2 or early L3 stage from each strain shown. Relative *lin-3* mRNA levels were normalized to the levels of mRNA encoding the ribosomal protein subunit *rpl-26* using the ΔΔCt method [52]. The means and standard deviations of relative *lin-3* mRNA levels from two independent trials are shown. The *lin-15A(n767)*, *lin-15B(n744)* and *lin-15AB(e1763)* alleles were used in this experiment. (C) *F36H1.12* mRNA levels in synMuv single and double mutants. No combination of *lin-3(n4441)*, *lin-15A*, and *lin-15B* mutations affected *F36H1.12* mRNA levels. Realtime RT-PCR experiments were performed using RNA harvested at the late L2 or early L3 stage from each strain shown. Relative *F36H1.12* mRNA levels were normalized to the levels of mRNA encoding the ribosomal protein subunit *rpl-26* using the ΔΔCt method. The means and standard deviations of relative *F36H1.12* mRNA levels from two independent trials are shown.

The class A and B synMuv genes redundantly repress expression of *lin-3* mRNA [27]. To test if the *lin-3(n4441)* mutation affects *lin-3* mRNA levels similarly to other class A synMuv mutations, we assayed *lin-3* mRNA levels using real-time RT-PCR. As previously reported, the class B synMuv mutant *lin-15B(n744)* has wild-type *lin-3* levels (Figure 2B). The class A synMuv mutants *lin-15A(n767)* and *lin-3(n4441)* both had slightly increased levels of *lin-3* mRNA. The synMuv double mutants *lin-15AB(e1763)* and *lin-3(n4441); lin-15B(n744)* had substantially increased *lin-3* mRNA levels (Figure 2B). Therefore, the *lin-3(n4441)* mutation behaves as a class A synMuv mutation with respect to *lin-3* mRNA repression.

The *lin-3(n4441)* mutation is located 211 bp upstream of *lin-3* and is also 465 bp upstream of *F36H1.12*, which is upstream of *lin-3* in the opposite orientation (Figure 2A). To determine if *lin-3(n4441)* or other synMuv mutations also affect expression of *F36H1.12*, we assayed *F36H1.12* mRNA levels by real-time RT-PCR. *F36H1.12* mRNA levels were roughly equivalent to those of the wild type in all possible single and double mutant combinations involving *lin-15A(n767)*, *lin-3(n4441)*, and *lin-15B(n744)* (Figure 2C). Therefore, the synMuv proteins specifically repress *lin-3* and do not establish a broad domain of repression.

### Expression pattern of *lin-3* in wild-type animals and synMuv mutants

Although *lin-3* is overexpressed in synMuv double mutants [27] (also, Figure 2), it is not known where this overexpression occurs. GFP- and LacZ-tagged *lin-3* repetitive transgene arrays have been used as reporters for *lin-3* expression [10], [39], [40], but these reporters might not be appropriate for determining *lin-3* expression in synMuv mutants: first, the level of ectopic *lin-3* expression might be too low to visualize using a GFP reporter; second, many synMuv mutations affect the expression of repetitive transgene arrays, potentially confounding interpretation of the expression pattern of such reporters [41]. Instead, we assayed *lin-3* expression using a fluorescence *in situ* hybridization (FISH) technique that has sufficient sensitivity to detect single mRNA molecules [42]. We used 48 non-overlapping probes against *lin-3* (Table S1), each conjugated to a single fluorophore, to label individual mRNA molecules brightly enough to be visible as distinct fluorescent spots. Because there are 48 probes that bind independently to the target mRNA, any single probe that binds non-specifically should not cause a false-positive signal. The distribution of intensities of the spots in any given animal was unimodal, consistent with each spot's representing a single mRNA molecule (Figure S1). Furthermore, by comparing the spot intensities in different tissues and mutants, we found that the level of expression in a given cell or tissue was independent of the intensity of the spots in that cell or tissue, and if the number of spots in a cell was altered then the average intensity of spots in that cell was unchanged (Figure S2). If each spot represented multiple mRNA molecules, then as the expression level in a given cell increased the average number of mRNA molecules in each of those spots would also be expected to increase, leading to greater intensity. Because the intensity of each spot was independent of the level of expression, we conclude that each spot is likely to represent a single mRNA molecule. We also found that all tissues are accessible to FISH probes, as probes directed against *ama-1* and *eft-2* robustly detected mRNA in all cells (data not shown). However, we cannot know if we are detecting every mRNA molecule; it is possible that some mRNA molecules are not accessible to the oligonucleotide probes or are not detected for some other reason.

**Figure 3:**
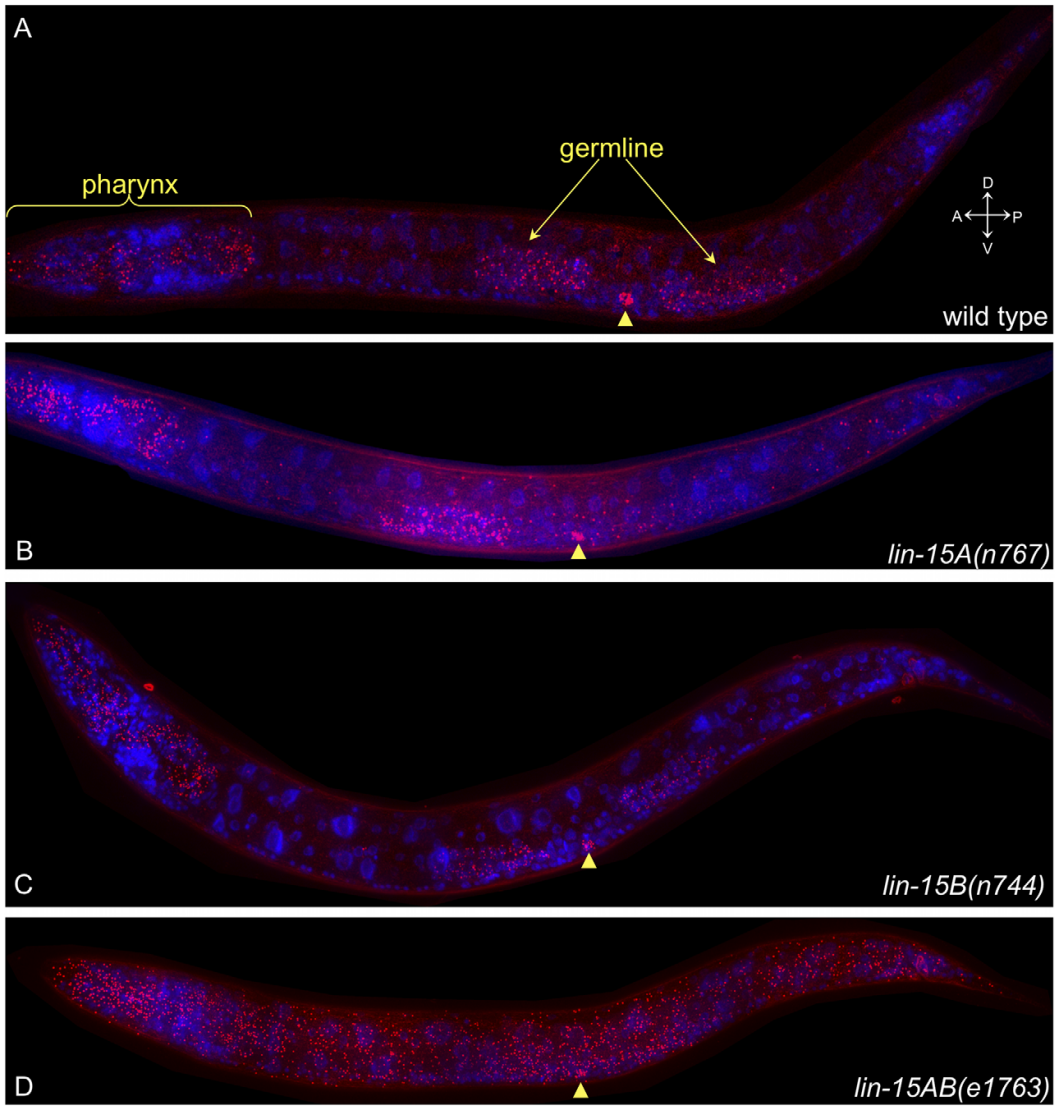
The synMuv genes prevent widespread ectopic expression of *lin-3* mRNA. FISH of *lin-3* mRNA in late L2 to early L3 animals. Each dot represents a single mRNA molecule [42]. *lin-3* mRNAs are shown in red, and 4′,6-diamidino-2-phenylindole (DAPI) staining of nuclei is shown in blue. The images shown are maximum intensity projections of a z-stack of images. The anchor cell (AC) is indicated by an arrowhead in each panel. (A) Wild type. *lin-3* mRNA is expressed in the pharynx, anchor cell, and germline and is tightly repressed elsewhere. (B) *lin-15A(n767)*. There is a low level of ectopic *lin-3* expression, with approximately 60 ectopic copies of *lin-3* mRNA seen outside the pharynx, anchor cell, and germline. (C) *lin-15B(n744)*. There is a very low level of ectopic *lin-3* expression, with approximately 10 ectopic copies of *lin-3* mRNA seen outside the pharynx, anchor cell, and germline. The large bright spot at the edge of the head is outside the animal. (D) *lin-15AB(e1763)*. *lin-3* is ectopically expressed throughout the animal, with approximately 900 ectopic copies of *lin-3* mRNA seen outside the pharynx, anchor cell, and germline.

We first determined the expression pattern of *lin-3* in wild-type animals at the late L2 to early L3 stage when vulval induction occurs. Previous studies found that at the early L3 stage *lin-3* is expressed in the anchor cell and in the pharynx [10], [40]. We indeed observed robust expression of *lin-3* in the anchor cell and throughout the pharynx. We also saw expression of *lin-3* in the germline (Figure 3A and Figure S3). In some wild-type animals we also observed a few copies of *lin-3* mRNA in one or more cells in the tail, on the ventral side slightly anterior to the anus. In addition, a few copies of *lin-3* mRNA were seen on the ventral side of the animal, slightly behind the posterior gonad arm. We imaged several animals that were slightly older, in the late L3 stage, and observed expression of several copies of *lin-3* mRNA in the region where P6.p and its descendants are located (data not shown), consistent with previous reports of expression of *lin-3* in the descendants of P6.p by the L4 stage [39]. We did not consistently detect any *lin-3* mRNA in other tissues, although in some animals we observed a single *lin-3* mRNA molecule elsewhere. For example, in the animal shown in Figure 3A a single *lin-3* mRNA molecule was observed in or near an intestinal cell close to the anchor cell. Overall, other than for those tissues that highly expressed *lin-3* there was very tight repression of *lin-3*. The numbers of copies of *lin-3* mRNA we observed in each tissue in individual animals are listed in Table S2 and are summarized in Table 3. The expression pattern we observed for *lin-3* is consistent with that seen using GFP- and LacZ-tagged *lin-3* reporters [10], [39], [40] and with functional studies of *lin-3* [43], indicating that most if not all of the mRNA spots identified by this technique are likely to represent actual *lin-3* mRNA molecules.

**Table 3:** Quantification of *lin-3* expression.

genotype	n^a^	Anchor cell	Germline	Pharynx	Leaky^b^
N2	6	29±5	314±69	284±48	1±1
*lin-15A(n767)*	7	23±5	279±46	451±92	64±21
*lin-15B(n744)*	6	21±4	199±57	380±134	6±2
*lin-15AB(e1763)*	7	22±4	231±54	503±154	1087±211
*lin-3(n4441)*	8	26±4	340±62	387±68	71±28
*lin-3(n4441); lin-15B(n744)*	5	21±7	360±109	578±70	1156±111

The average number of copies of *lin-3* mRNA observed in each tissue and the standard deviation between animals is shown.

a n, total number of animals assayed.

b *lin-3* mRNA observed outside of the normal domain of expression of *lin-3*.

*lin-15AB(e1763)* animals expressed *lin-3* in the pharynx, germline, and anchor cell at levels grossly similar to those of wild-type animals (Figure 3D and Table 3). In addition there was widespread ectopic expression of *lin-3*, with an average of approximately 1100 ectopic copies of *lin-3* mRNA observed per animal (Figure 3D and Table 3). This ectopic expression was much weaker than the normal expression in the anchor cell; whereas an average of 29 copies of *lin-3* mRNA was seen in the anchor cell in wild-type animals (Table 3), only one or a few copies of *lin-3* mRNA were observed in most cells in *lin-15AB(e1763)* mutants. Because we could not see cell boundaries, we could not determine if every cell ectopically expressed *lin-3*, but there were no tissues that appeared to lack ectopic *lin-3* mRNA (Figure S4). Cells around the perimeter of the animal expressed *lin-3* in the *lin-15AB(e1763)* mutant, consistent with ectopic expression in the hypodermis (Figure 3D and Figure S4). There were also many ectopic *lin-3* mRNA copies that clearly were not in the hypodermis (Figure S4).

We also determined *lin-3* expression in *lin-15A(n767)* and *lin-15B(n744)* single mutants. *lin-15B(n744)* animals had a *lin-3* expression pattern similar to that of wild-type animals (Figure 3C). In *lin-15B(n744)* mutants there was an extremely low level of ectopic *lin-3* expression, with an average of six ectopic *lin-3* mRNA molecules detected per animal (Table 3), but *lin-3* was still tightly repressed outside of the germline, anchor cell, and pharynx. *lin-15A(n767)* animals exhibited broad ectopic expression of *lin-3*, but at a much lower level than that of *lin-15AB(e1763)* animals (Figure 3B). An average of 64 copies of *lin-3* mRNA were seen outside of the pharynx, germline, and anchor cell in *lin-15A(n767)* animals (Table 3). Unlike in *lin-15AB(e1763)* animals, in any given *lin-15A(n767)* animal most cells did not display ectopic *lin-3* expression. However, we observed no obvious cell or tissue specificity to the ectopic expression among several *lin-15A(n767)* animals. Rather, it appeared that in *lin-15A(n767)* animals *lin-3* is globally derepressed, but at a very low level.

**Figure 4:**
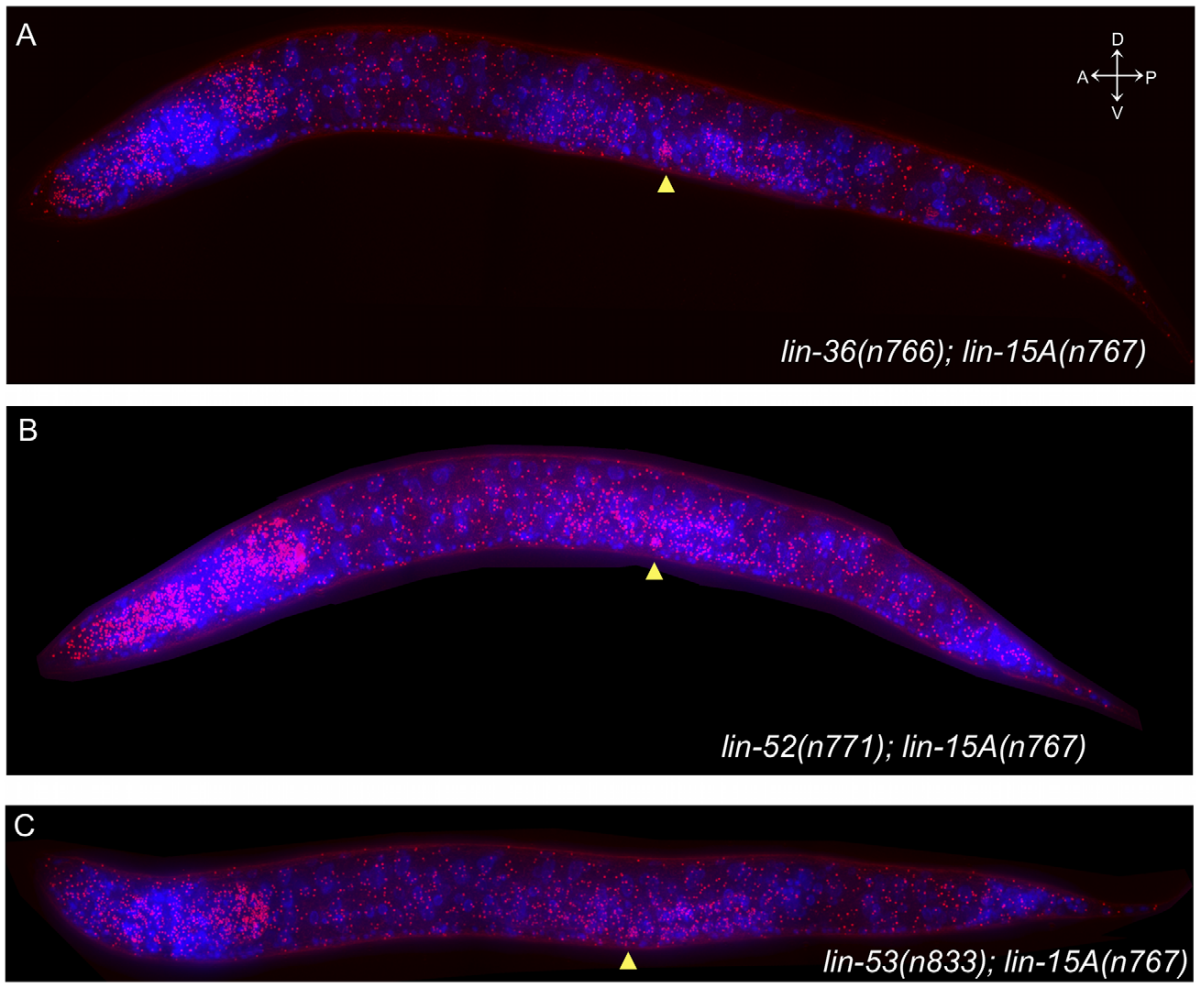
Multiple class B synMuv mutations have similar effects on *lin-3* mRNA expression. FISH of *lin-3* mRNA in late L2 to early L3 animals. Each dot represents a single mRNA molecule [42]. *lin-3* mRNAs are shown in red, and 4′,6-diamidino-2-phenylindole (DAPI) staining of nuclei is shown in blue. The images shown are maximum intensity projections of a z-stack of images. The anchor cell (AC) is indicated by an arrowhead in each panel. Each mutant displayed ectopic *lin-3* mRNA expression throughout the animal in most if not all tissues. (A) *lin-36(n766); lin-15A(n767)*. (B) *lin-52(n771); lin-15A(n767)*. (C) *lin-53(n833); lin-15A(n767)*.

The numerous class B synMuv genes have highly similar although not identical effects on vulval development [15]. However, the class B synMuv genes have widely differing effects on other aspects of growth and development. For example, PGL-1, which is normally expressed in the germline, is misexpressed in the somatic cells of mutants of many class B synMuv genes, including *lin-15B*, but not in mutants of some other class B synMuv genes, including *lin-36*, *lin-52*, and *lin-53* [44], [45]. We therefore investigated the role of the class B synMuv genes *lin-36*, *lin-52*, and *lin-53* in controlling *lin-3* expression. We determined the expression pattern of *lin-3* in *lin-36(n766)*; *lin-15A(n767)*, *lin-52(n771); lin-15A(n767)*, and *lin-53(n833); lin-15A(n767)* mutants. All three double mutants exhibited ubiquitous ectopic expression of *lin-3*, with *lin-3* mRNA observed in most if not all tissues (Figure 4). There were no obvious differences in the spatial pattern of *lin-3* expression in *lin-36(n766)*; *lin-15A(n767)*, *lin-52(n771); lin-15A(n767)*, and *lin-53(n833); lin-15A(n767)* double mutants as compared to *lin-15AB(e1763)* mutants.

**Figure 5:**
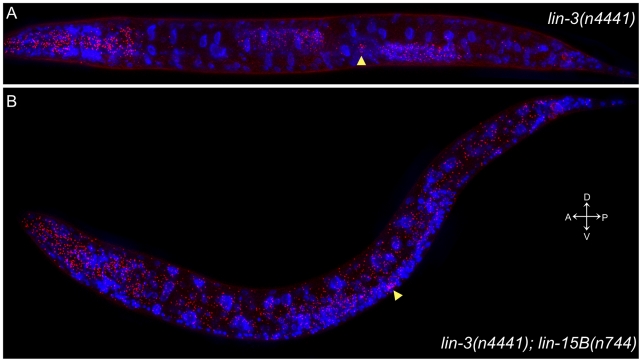
The *lin-3(n4441)* mutation causes widespread ectopic expression of *lin-3* mRNA. FISH of *lin-3* mRNA in late L2 to early L3 animals. Each dot represents a single mRNA molecule [42]. *lin-3* mRNAs are shown in red, and 4′,6-diamidino-2-phenylindole (DAPI) staining of nuclei is shown in blue. The images shown are maximum intensity projections of a z-stack of images. The anchor cell (AC) is indicated by an arrowhead in each panel. (A) *lin-3(n4441)*. there is a low level of ectopic *lin-3* expression, with approximately 45 ectopic copies of *lin-3* mRNA seen outside the pharynx, anchor cell, and germline. (B) *lin-3(n4441); lin-15B(n744)*. *lin-3* is ectopically expressed throughout the animal, with approximately 1000 ectopic copies of *lin-3* mRNA seen outside the pharynx, anchor cell, and germline.

The *lin-3(n4441)* mutation could cause global derepression of *lin-3* similarly to *lin-15A(n767)*, or it could affect *lin-3* expression in a subset of tissues. We examined the expression of *lin-3* mRNA in *lin-3(n4441)* and *lin-3(n4441); lin-15B(n744)* animals. *lin-3(n4441)* animals had widespread but weak ectopic expression of *lin-3*, similar to *lin-15A(n767)* animals (Figure 5A and Table 3). *lin-3(n4441); lin-15B(n744)* animals exhibited ectopic *lin-3* expression in most cells and were indistinguishable from *lin-15AB(e1763)* animals (Figure 5B and Table 3).

## Discussion

Identifying the biologically relevant targets of transcriptional regulators that control development is a challenging problem. The synMuv genes encode putative transcriptional repressors that prevent ectopic vulval development. Mutating a synMuv binding site in a target gene might relieve repression of that target, and if that repression were essential to prevent ectopic vulval development could cause a dominant synMuv phenotype. We isolated a mutation in the *lin-3* EGF gene that derepresses *lin-3* transcription and causes a dominant class A synMuv phenotype. This finding establishes that *lin-3* is a functionally important target of the class A synMuv genes, consistent with a previous report that *lin-3* expression is repressed by the synMuv genes and that double-stranded RNA directed against *lin-3* can suppress the synMuv phenotype [27]. Importantly, the *lin-3(n4441)* mutation fully recapitulates the class A synMuv phenotype with regard to vulval development and *lin-3* expression and causes a class A synMuv phenotype equivalent to that caused by strong alleles of class A synMuv genes. If the class A synMuv genes repressed multiple targets to prevent ectopic vulval development, then a mutation that abolished class A synMuv-mediated repression of *lin-3* would recapitulate only partially the class A synMuv phenotype. We conclude that *lin-3* is likely to be the only key biologically relevant target of the class A synMuv genes in vulval development.

The simplest interpretation of the effect of the *lin-3(n4441)* mutation is that this mutation abolishes a binding site for a transcriptional repressor consisting of or controlled by class A synMuv proteins. However, the effect of the *lin-3(n4441)* mutation is slightly enhanced by mutations in all other class A synMuv genes. If the *lin-3(n4441)* mutation completely inactivated a binding site that responds to only one of the known class A synMuv proteins, then mutation of that class A synMuv gene should not enhance the synMuv phenotype caused by *lin-3(n4441)*. One possibility is that a complex consisting of multiple class A synMuv proteins binds to the *lin-3* promoter, the *lin-3(n4441)* mutation strongly reduces but does not completely eliminate that binding, and removing any one class A synMuv protein does not fully abrogate the ability of the complex to bind to the *lin-3* locus and repress transcription. This model is consistent with the observation that most class A synMuv mutations, including *lin-3(n4441)*, are enhanced by class A synMuv mutations in other genes [15]. Alternatively, the class A synMuv genes might indirectly repress *lin-3* by regulating the expression or activity of or by binding to another protein that binds to the *lin-3* promoter to prevent ectopic transcription. Because a mutation in the *lin-3* promoter can cause a class A synMuv phenotype, the class A and class B synMuv pathways must be integrated at the point of *lin-3* repression, and hence it is unlikely that the class A and B synMuv genes redundantly control a transcriptional regulator which in turn controls *lin-3* expression.

*lin-3* expression in the germline had not been previously observed, likely because the reporters used to assay *lin-3* expression were either silenced in the germline [46] or lacked distant regulatory regions necessary to drive germline expression. Mutations in the FOG and FBF translational inhibitor RNA-binding proteins cause a germline-dependent Muv phenotype, and the FBF proteins can bind to the 3′ UTR of *lin-3 in vitro*, suggesting that germline *lin-3* mRNA is translationally repressed during the larval stage when vulval induction occurs [43]. In many class B synMuv mutants, somatic cells express normally germline-specific genes [19], [44], [45]. Given our finding that *lin-3* is normally expressed in the germline, one possibility is that the class B synMuv genes repress ectopic *lin-3* expression in somatic cells as a consequence of their role in ensuring that somatic cells do not inappropriately adopt germline-like fates. The class B synMuv genes might all directly repress *lin-3* in somatic cells. Alternatively, as there are a large number of class B synMuv genes and their effects on vulval development are not identical, perhaps at least some class B synMuv genes indirectly repress *lin-3* by preventing the ectopic adoption of germline-like fates. In class B synMuv single mutants, the somatic cells adopt a more germline-like fate that would include *lin-3* expression except that the class A synMuv genes still tightly repress *lin-3*, mostly preventing ectopic *lin-3* expression. In class A synMuv single mutants, *lin-3* is not tightly repressed, but most somatic cells are not fated to express *lin-3*, so there is only a low level of leaky ectopic *lin-3* expression. However, in class AB synMuv double mutants, the somatic cells adopt a germline-like fate that includes *lin-3* expression, and there is no class A synMuv mechanism that tightly represses *lin-3*, resulting in widespread and substantial ectopic *lin-3* expression. In short, we suggest that the synthetic Muv phenotype caused by mutations in the synMuv genes might be a consequence of two distinct functions of the class A and class B synMuv genes: the class A synMuv genes either directly or indirectly tightly repress ectopic *lin-3* transcription, and the class B synMuv genes prevent somatic expression of germline-expressed genes, which include *lin-3*; only if both functions are lost will somatic cells ectopically express sufficient *lin-3* mRNA to cause ectopic vulval induction. These findings raise the possibility that the development of some human tumors might require the loss of one tumor suppressor gene that prevents cells from adopting a fate that is permissive for the expression of a growth factor and the loss of a second tumor suppressor gene that specifically represses the expression of that growth factor.

A subset of the class B synMuv genes is required to prevent the somatic misexpression of normally germline-restricted P-granule proteins such as PGL-1 [44], [45]. We found that *lin-15B* mutants, which do exhibit somatic PGL-1 expression, and *lin-36*, *lin-52*, and *lin-53* mutants, which do not exhibit somatic PGL-1, all have highly similar effects on *lin-3* expression. These results indicate that different germline genes are broadly repressed in the soma by different sets of transcriptional repressors. The class B synMuv genes define one such group of repressors and are classified together because they have comparable effects on the germline gene *lin-3*, resulting in similar vulval phenotypes. Many such partially-overlapping groups of transcriptional repressors, including various subsets of the class B synMuv genes, are likely to be required for the repression in the soma of other germline-restricted genes.

Whereas *lin-3* expression in most cells is tightly repressed by the synMuv genes, the anchor cell and germline exhibit robust *lin-3* expression that is not substantially affected by the synMuv genes. While it has not been reported whether or not any synMuv genes are expressed in the anchor cell, several synMuv genes are expressed in the germline [17], [22], [26], [33], and we are not aware of studies that have conclusively shown any synMuv genes not to be expressed in the germline. In most cells, the synMuv genes reduce *lin-3* expression from an average of one to two copies per cell to nearly zero copies per cell. The synMuv genes clearly do not have a similar fold effect on *lin-3* expression in the anchor cell and germline. However, it is possible that the synMuv genes repress a similar absolute number of leaky *lin-3* mRNA molecules in all cells; given the animal-to-animal variability in *lin-3* expression we likely would not have been able to detect such a small increase in the anchor cell or germline. Alternatively, the synMuv genes might not repress *lin-3* in the anchor cell or germline. The strong activator(s) of *lin-3* that drive expression in those tissues could override the activity of the synMuv genes, or one or more synMuv genes might not be expressed in those tissues, thereby compromising synMuv repression of *lin-3*.

In *lin-15AB* mutants, *lin-3* is ectopically expressed throughout the animal in a broad range of cells and tissues. Site-of-action experiments have shown that the synMuv genes function at least in large part in the hyp7 hypodermal syncytium to prevent ectopic vulval development [31]. The expression pattern of *lin-3* in synMuv mutants does not directly identify the site-of-action of synMuv genes in regulating vulval development but does show that the synMuv genes function throughout the animals to keep *lin-3* very tightly repressed in numerous cells and tissues. *lin-3* EGF regulates non-vulval cell fates in *C. elegans* development, and at least some of these fates, such as the P11/P12 fate, are also regulated by the synMuv genes in a manner analogous to that of vulval development [47]. In short, the synMuv genes act throughout the animal to prevent ectopic *lin-3* expression, which can cause a variety of developmental abnormalities. Mutants with a displaced anchor cell show that *lin-3* can act at a distance [48], so a cell ectopically expressing *lin-3* could affect fates in both nearby and distant cells. We suggest that for any given cell-fate decision, the site of action of the synMuv genes is likely to be spread across multiple cells and determined by the size and proximity of those cells to the cell being regulated by *lin-3*. In the case of vulval development, hyp7 plays the major role, given its large size and close proximity to the Pn.p cells, with likely lesser contributions from many other cells. The site at which the synMuv genes repress *lin-3* to ensure proper vulval development is therefore probably a combination of the Pn.p cells themselves and neighboring cells that do not normally either express or respond to *lin-3*. This situation is similar to that in which both tumor cells and the microenvironment surrounding the tumor provide factors that drive tumor development [49]. We suggest that analogously to the synMuv genes some tumor suppressor genes function by repressing growth factor expression in both tumor cells and the surrounding microenvironment.

In synMuv double mutants, *lin-3* was ectopically expressed but at a much lower level than at its major normal site of function, the anchor cell. synMuv double mutants might ectopically express as few as one to two copies of *lin-3* mRNA per cell. Thus, normal *C. elegans* development requires *lin-3* to be exceedingly tightly repressed outside of a few cells, and only slight expression of *lin-3* throughout the animal can cause abnormal cell-fate transformations. Such low levels of ectopic expression would likely be missed by most techniques used to assay gene expression. We suggest it could be important to examine the expression of EGF-family ligands in tumors using highly sensitive techniques with single-molecule resolution to determine if broad low-level misexpression of EGF-family ligands plays a role in oncogenic growth. In *C. elegans*, the tight repression of *lin-3* EGF requires both the class A synMuv gene pathway and the class B gene synMuv pathway, which includes homologs of known tumor suppressor genes, such as *lin-35* Rb. Therefore, some tumor suppressor genes in mammals might function by tightly repressing low-level ectopic expression of EGF-family ligands in many cells, possibly in both the tumor and the microenvironment surrounding the tumor.

## Materials and Methods

### Strains and genetics

*C. elegans* strains were cultured by standard methods on OP50 bacteria [50]. All animals were grown at 20°C, except where otherwise noted. The wild-type strain was N2, except in SNP mapping experiments in which the polymorphic CB4856 strain was also used [51]. The following mutations were used in this study:

LGI: *dpy-5(e61)*, *lin-61(n3447)*, *lin-53(n833)*

LGII: *lin-8(n2731)*, *lin-56(n2728)*, *lin-38(n751)*, *syIs12*

LGIII: *dpy-17(e164)*, *lin-36(n766)*, *unc-32(e189)*, *lin-52(n771)*

LGIV: *lin-3(n4441)*, *lin-3(n4929)*, *lin-3(n4951)*, *lin-3(n1059)*

LGX: *lin-15A(n767)*, *lin-15B(n744)*, *lin-15(e1763)*

The balancer strain *nT1[qIs51]* IV∶V [34] was used; *qIs51* is a GFP-expressing transgene integrated onto the *nT1* translocation. Table S3 lists all strains used in this study.

### Quantitative PCR assay

Synchronized animals were harvested at or near the L2-to-L3 larval transition, when vulval induction occurs. N2 animals were harvested 33 hours after starved L1 larvae were placed on plates with food. Some mutants grew more slowly and were harvested after 39 hours. Quantitative RT-PCR for *lin-3* was performed as previously described [15]. *lin-3* was amplified using the primers CGCATTTCTCATTGTCATGC and CTGGTGGGCACATATGACTC.

### Fluorescence *in situ* hybridization

Animals were grown to the L2-to-L3 transition as in the quantitative RT-PCR experiments. Fixation and hybridization were performed as described previously [42], except that worms were fixed for one hour instead of 45 minutes. The *lin-3* probes (Biosearch Technologies, Inc) were conjugated to the fluorophore Cy5 using the Amersham Cy5 Mono-reactive Dye pack (GE Healthcare). DNA was visualized using 4′,6-diamidino-2-phenylindole (DAPI). The probe sequences used are shown in Table S1. Figure 2 and Figure 3 are maximum intensity projections of a Z-stack of images processed with the Find Edges and Smooth operations in ImageJ. *lin-3* mRNA spots were computationally identified with manually determined thresholds as previously described [42]. The number of molecules within each tissue were then manually counted. The anchor cell was identified based on position. *lin-3* mRNA expression in the anchor cell appeared as a tight cluster of spots; molecules within that cluster were considered to be in the anchor cell. To determine which *lin-3* mRNA molecules were in the pharynx, we noted the outline of the pharynx that was clearly visible as a dark boundary in the Cy5 channel of the image stacks (see Figure S3 and Figure S4). The boundaries of the germline were estimated from the positions of DAPI-labeled germline nuclei.

## Supporting Information

Figure S1 Unimodal distribution of FISH spot intensity. Histograms of FISH spot intensity for six images from six different animals are shown. Each image included the anchor cell and most or all of the germline. Intensities were calculated by taking the maximum intensity of a spot and subtracting the average background intensity of a four-pixel radius surrounding the spot.
(TIFF)

Figure S2 FISH spot intensity is independent of expression level. The mean spot intensity was calculated for mRNAs expressed in the anchor cell, germline, or ectopically. For each animal, the intensity of each spot was normalized to the mean intensity of the spots in the anchor cell, which was set to 1. The mean and standard deviation for the expression in the germline or ectopically from 7–10 animals for each genotype are shown. *lin-3(e1417)* had roughly wild-type numbers of *lin-3* FISH spots in the germline but had approximately 4.5-fold fewer *lin-3* FISH spots in the anchor cell (Table S2). In both wild-type and *lin-15AB(e1763)* animals the density of spots in the anchor cell was noticeably higher than in the germline or elsewhere (e.g. Figure 3A and 3D).
(TIFF)

Figure S3 *lin-3* mRNA expression in a wild-type animal. FISH of *lin-3* mRNA in a wild-type animal. The animal shown is the same as in Figure 3A. Each frame is a different plane in the Z-axis. *lin-3* mRNA was expressed in the pharynx, anchor cell, and germline and was tightly repressed elsewhere.
(AVI)

Figure S4 The synMuv genes prevent widespread ectopic expression of *lin-3* mRNA. FISH of *lin-3* mRNA in a *lin-15AB(e1763)* mutant animal. The animal shown is the same as in Figure 3D. Each frame is a different plane in the Z-axis. *lin-3* was ectopically expressed throughout the animal, with approximately 900 ectopic copies of *lin-3* mRNA seen outside of the pharynx, anchor cell, and germline.
(AVI)

Table S1 Oligonucleotides in *lin-3* FISH probe.
(DOC)

Table S2 Quantification of *lin-3* expression.
(DOC)

Table S3 List of strains used in this study.
(DOC)
