# Supplementary figures and images for: The Caenorhabditis elegans Synthetic Multivulva Genes Prevent Ras Pathway Activation by Tightly Repressing Global Ectopic Expression of lin-3 EGF
Source: PLoS Genet. 2011 Dec 29;7(12):e1002418. doi: 10.1371/journal.pgen.1002418 (PMC3248470; doi:10.1371/journal.pgen.1002418)

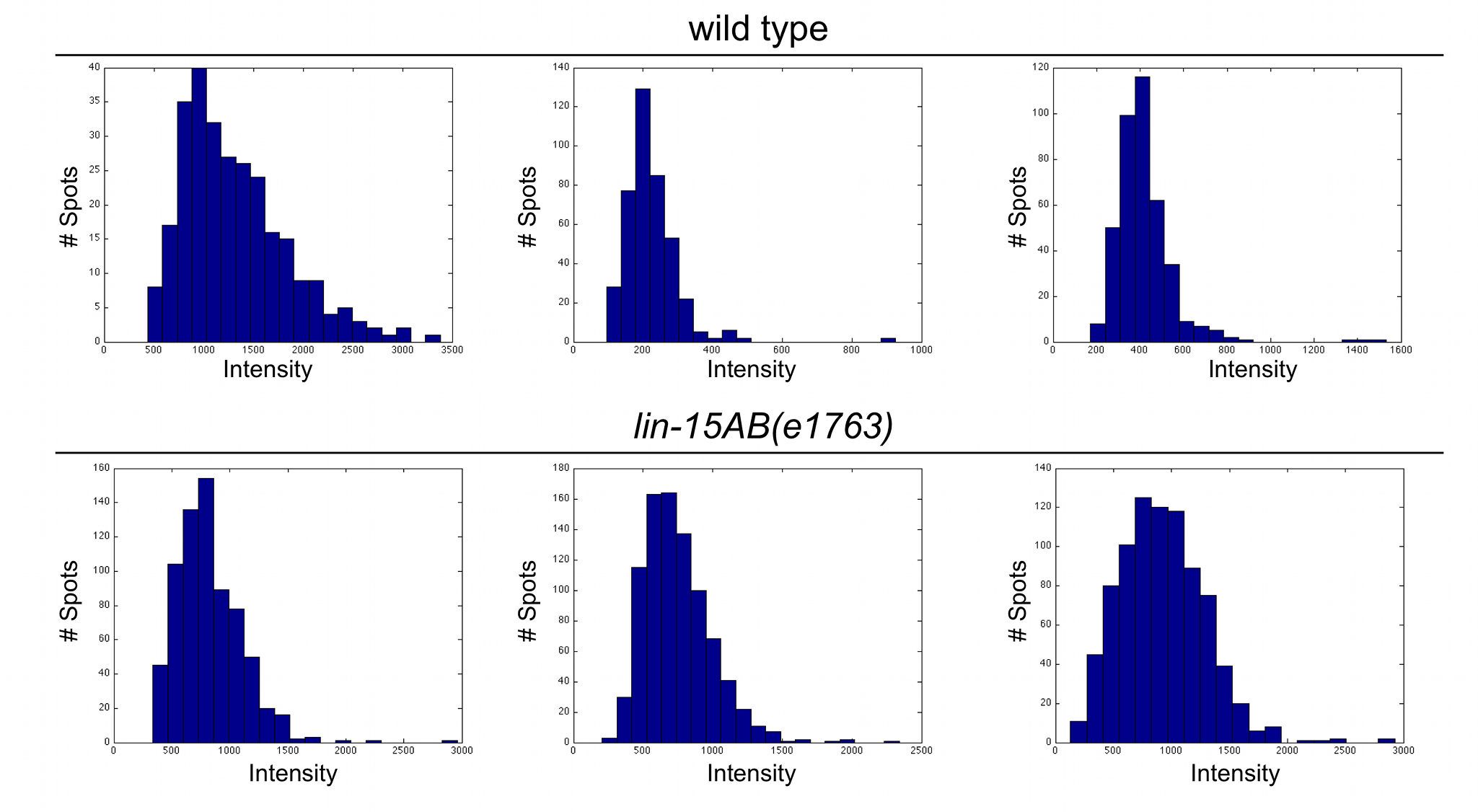

Supplement: Figure S1 — Unimodal distribution of FISH spot intensity. Histograms of FISH spot intensity for six images from six different animals are shown. Each image included the anchor cell and most or all of the germline. Intensities were calculated by taking the maximum intensity of a spot and subtracting the average background intensity of a four-pixel radius surrounding the spot. (TIFF) [file pgen.1002418.s001.tiff]

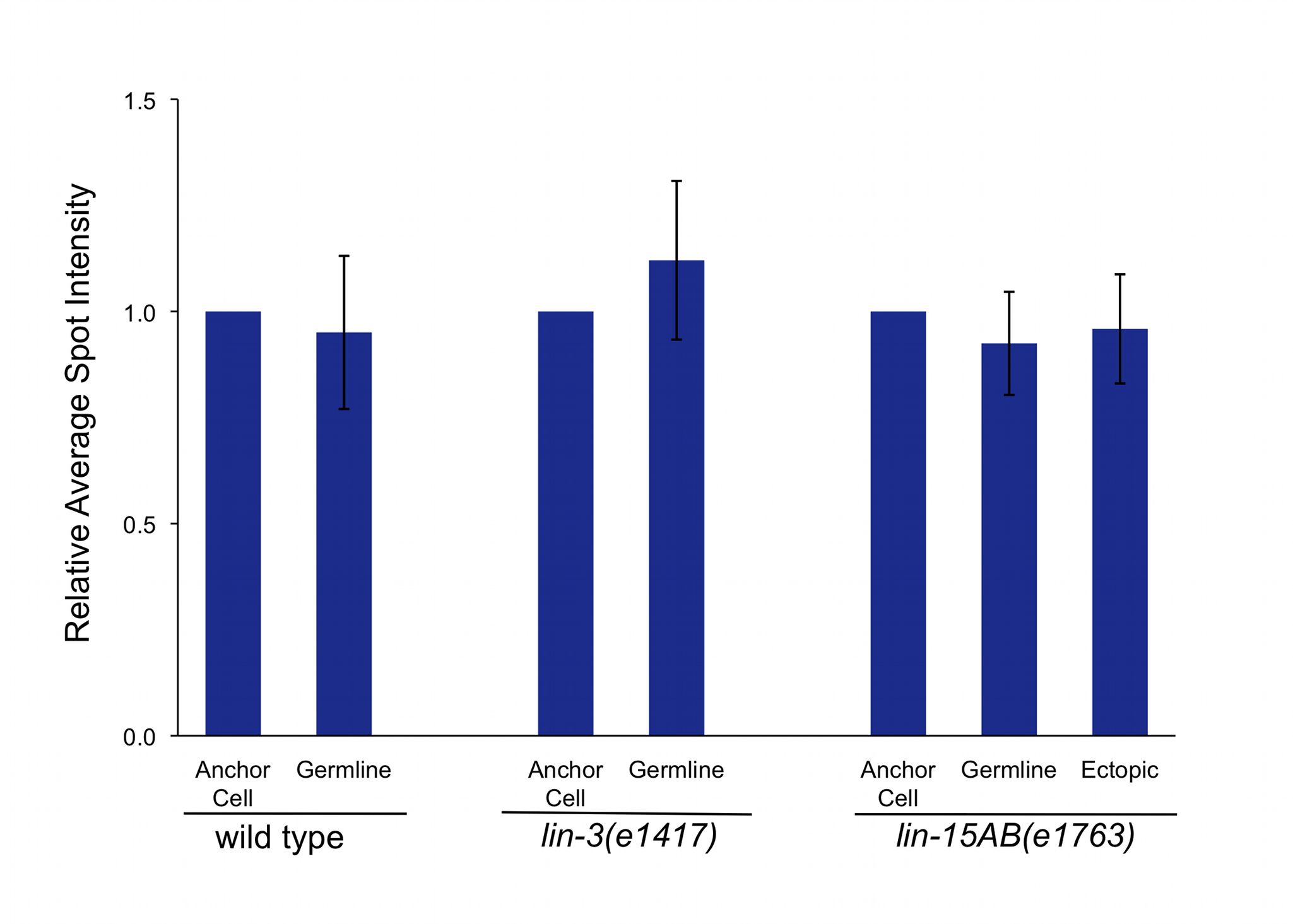

Supplement: Figure S2 — FISH spot intensity is independent of expression level. The mean spot intensity was calculated for mRNAs expressed in the anchor cell, germline, or ectopically. For each animal, the intensity of each spot was normalized to the mean intensity of the spots in the anchor cell, which was set to 1. The mean and standard deviation for the expression in the germline or ectopically from 7–10 animals for each genotype are shown. lin-3(e1417) had roughly wild-type numbers of lin-3 FISH spots in the germline but had approximately 4.5-fold fewer lin-3 FISH spots in the anchor cell (Table S2). In both wild-type and lin-15AB(e1763) animals the density of spots in the anchor cell was noticeably higher than in the germline or elsewhere (e.g. Figure 3A and 3D). (TIFF) [file pgen.1002418.s002.tiff]
