# Supplementary material for: The Caenorhabditis elegans Synthetic Multivulva Genes Prevent Ras Pathway Activation by Tightly Repressing Global Ectopic Expression of lin-3 EGF
Source: PLoS Genet. 2011 Dec 29;7(12):e1002418. doi: 10.1371/journal.pgen.1002418 (PMC3248470; doi:10.1371/journal.pgen.1002418)
Supplement: Table S1 — Oligonucleotides in lin-3 FISH probe. (DOC) [file pgen.1002418.s005.doc]

| Table S1: Oligonucleotides in *lin-3* FISH probe | |
| --- | --- |
| caagccgacgatccgttatt | tatatctgccgttgattggg |
| aattgatgcataaggggtgg | tcatcgtgctcaaacgtaca |
| gcgacgatatcttatgaaac | tttccttgaacgaggagttg |
| ccgcattttggaaacttgtg | ggcgttgtagaaatcgtttg |
| gaggcataaagagtagaagg | ctctcataaagcccactgaa |
| gggagacacgattctgtaaa | gctgatgttgaagattgcac |
| tacgttcttgacgaaaccac | agaaatgcgaacgcaggaat |
| tctgcagattgaagctgttc | gggcacatatgactcattgt |
| gccactattttcagctgcat | aggacattgaatgcttctgg |
| atttcgagaagtatcgggtg | ggaatatgtcgtccatttgg |
| ggtgcatcacctatttcgtt | cggtgttgggatagtataag |
| taggtgtttcaggtgtcgaa | cttgatccaggagttgatga |
| gcttcggaaatcgtagtttc | aatagcttgttgacgagtgg |
| gttcgtttttcatcgtctcc | gttgttccgtgcttgttcat |
| caccctcatattctgcttct | cttgacttctgagaatgctg |
| cttctacttcttcatcaacc | tactcctggatggaatggta |
| catcttgagtggcatcttca | actgacttgtagtgcttcgg |
| ttctcgatttccttccgaac | tgctgaaacttcaacacgtg |
| gtgatgacagtagtctttgc | cttctgattcagtcgactga |
| atatcacttccacgtggcat | gtggacatgttactgatgct |
| ctgaaactctatcttcacgg | attaattacagtgtgcgccg |
| caatggcaagaaggaacaac | atctgcagaatccaactcga |
| acgatcacaacgagtgcctt | tgttctccagaacttcgaga |
| atagaacgcctgaacgtagt | ttcacatgttgctggtgatc |
