# Supplementary material for: The Caenorhabditis elegans Synthetic Multivulva Genes Prevent Ras Pathway Activation by Tightly Repressing Global Ectopic Expression of lin-3 EGF
Source: PLoS Genet. 2011 Dec 29;7(12):e1002418. doi: 10.1371/journal.pgen.1002418 (PMC3248470; doi:10.1371/journal.pgen.1002418)
Supplement: Table S2 — Quantification of lin-3 expression. (DOC) [file pgen.1002418.s006.doc]

| **Table S2:** Quantification of *lin-3* expression | | | | | |
| --- | --- | --- | --- | --- | --- |
| **Genotype** | **Anchor cell** | **Germline** | **Pharynx** | **Leaky^a^** | **Figure 3 or 5^b^** |
| N2 #1 | 23 | 253 | 193 | 0 |  |
| N2 #2 | 23 | 346 | 279 | 2 |  |
| N2 #3 | 33 | 205 | 324 | 1 | √ |
| N2 #4 | 28 | 341 | 288 | 2 |  |
| N2 #5 | 34 | 373 | 303 | 1 |  |
| N2 #6 | 30 | 367 | 315 | 0 |  |
| *lin-15A(n767)* #1 | 24 | 245 | 363 | 64 | √ |
| *lin-15A(n767)* #2 | 22 | 268 | 369 | 49 |  |
| *lin-15A(n767)* #3 | 17 | 272 | 522 | 55 |  |
| *lin-15A(n767)* #4 | 28 | 233 | 552 | 101 |  |
| *lin-15A(n767)* #5 | 17 | 249 | 332 | 52 |  |
| *lin-15A(n767)* #6 | 26 | 335 | 507 | 83 |  |
| *lin-15A(n767)* #7 | 28 | 352 | 514 | 41 |  |
| *lin-15B(n744)* #1 | 24 | 258 | 257 | 5 |  |
| *lin-15B(n744)* #2 | 21 | 192 | 478 | 10 | √ |
| *lin-15B(n744)* #3 | 20 | 177 | 272 | 6 |  |
| *lin-15B(n744)* #4 | 17 | 122 | 575 | 4 |  |
| *lin-15B(n744)* #5 | 27 | 174 | 430 | 4 |  |
| *lin-15B(n744)* #6 | 19 | 273 | 268 | 5 |  |
| *lin-15AB(e1763)* #1 | 28 | 274 | 603 | 710 |  |
| *lin-15AB(e1763)* #2 | 18 | 305 | 661 | 932 |  |
| *lin-15AB(e1763)* #3 | 27 | 218 | 674 | 1201 |  |
| *lin-15AB(e1763)* #4 | 20 | 265 | 416 | 1274 |  |
| *lin-15AB(e1763)* #5 | 17 | 191 | 345 | 1030 |  |
| *lin-15AB(e1763)* #6 | 23 | 216 | 292 | 1168 |  |
| *lin-15AB(e1763)* #7 | 23 | 147 | 530 | 1297 | √ |
| *lin-3(n4441)* #1 | 25 | 410 | 377 | 50 |  |
| *lin-3(n4441)* #2 | 21 | 381 | 397 | 68 | √ |
| *lin-3(n4441)* #3 | 24 | 335 | 298 | 46 |  |
| *lin-3(n4441)* #4 | 22 | 358 | 288 | 112 |  |
| *lin-3(n4441)* #5 | 34 | 414 | 402 | 114 |  |
| *lin-3(n4441)* #6 | 23 | 261 | 433 | 58 |  |
| *lin-3(n4441)* #7 | 27 | 263 | 408 | 48 |  |
| *lin-3(n4441)* #8 | 29 | 296 | 496 | 73 |  |
| *lin-3(n4441); lin-15B(n744)* #1 | 16 | 520 | 668 | 1112 |  |
| *lin-3(n4441); lin-15B(n744)* #2 | 22 | 267 | 541 | 1089 |  |
| *lin-3(n4441); lin-15B(n744)* #3 | 32 | 371 | 495 | 1322 | √ |
| *lin-3(n4441); lin-15B(n744)* #4 | 22 | 391 | 555 | 1209 |  |
| *lin-3(n4441); lin-15B(n744)* #5 | 14 | 349 | 630 | 1046 |  |
| *lin-3(e1417)* #1 | 8 | 325 | 525 | 0 |  |
| *lin-3(e1417)* #2 | 1 | 350 | 176 | 1 |  |
| *lin-3(e1417)* #3 | 11 | 219 | 347 | 0 |  |
| *lin-3(e1417)* #4 | 8 | 228 | 210 | 2 |  |
| *lin-3(e1417)* #5 | 6 | 287 | 222 | 0 |  |
| *lin-3(e1417)* #6 | 5 | 224 | 251 | 3 |  |
| *lin-3(e1417)* #7 | 5 | 309 | 279 | 3 |  |

The number of copies of *lin-3* mRNA observed in each tissue is shown.

^a^ *lin-3* mRNA observed outside of the normal domain of expression of *lin-3*

^b^ The animals shown in Figures 3 and 5 are noted
