# Supplementary material for: The Caenorhabditis elegans Synthetic Multivulva Genes Prevent Ras Pathway Activation by Tightly Repressing Global Ectopic Expression of lin-3 EGF
Source: PLoS Genet. 2011 Dec 29;7(12):e1002418. doi: 10.1371/journal.pgen.1002418 (PMC3248470; doi:10.1371/journal.pgen.1002418)
Supplement: Table S3 — List of strains used in this study. (DOC) [file pgen.1002418.s007.doc]

| **Table S3:** List of strains used in this study | |
| --- | --- |
| **Genotype** | **Strain** |
| *lin-3(e1417)* | CB1417 |
| *lin-15AB(e1763)* | CB3278 |
| *lin-36(n766); lin-15A(n767)* | MT1643 |
| *lin-52(n771); lin-15A(n767)* | MT1645 |
| *lin-15A(n767)* | MT1806 |
| *lin-38(n751)* | MT1808 |
| *lin-15B(n744)* | MT2495 |
| *lin-53(n833); lin-15A(n767)* | MT8374 |
| *lin-52(n771)* | MT8839 |
| *lin-56(n2728)* | MT8842 |
| *lin-8(n2731)* | MT10591 |
| *mys-1(n3681)* | MT12615 |
| *lin-61(n3447)* | MT12775 |
| *lin-3(n4441); lin-52(n771)* | MT14400 |
| *dpy-17(e164) lin-3(n4441) unc-32(e189)* | MT15256 |
| *lin-3(n4441)* | MT15257 |
| *lin-3(n4441); lin-15B(n744)* | MT15258 |
| *n4929 n4441/nT1[qIs51]; n744* | MT16254 |
| *lin-3(n4441); lin-15A(n767)* | MT16329 |
| *n4951 n4441/nT1[qIs51]; n744* | MT16557 |
| *syIs12; lin-15B(n744)* | MT16602 |
| *syIs12; lin-15A(n767)* | MT16603 |
| *lin-8(n2731); lin-3(n4441)* | MT17228 |
| *lin-38(n751); lin-3(n4441)* | MT17229 |
| *lin-56(n2728); lin-3(n4441)* | MT17230 |
| *lin-61(n3447); lin-3(n4441)* | MT17241 |
| *dpy-5(e61); lin-3(n4441); lin-15B(n744)* | MT19711 |
| *mys-1(n3681); lin-3(n4441)* | MT20720 |
| *syIs12* | PS2037 |
